# Analysis of *Pseudomonas aeruginosa* Cell Envelope Proteome by Capture of Surface-Exposed Proteins on Activated Magnetic Nanoparticles

**DOI:** 10.1371/journal.pone.0051062

**Published:** 2012-11-30

**Authors:** Davide Vecchietti, Dario Di Silvestre, Matteo Miriani, Francesco Bonomi, Mauro Marengo, Alessandra Bragonzi, Lara Cova, Eleonora Franceschi, Pierluigi Mauri, Giovanni Bertoni

**Affiliations:** 1 Department of Life Sciences, Università degli Studi di Milano, Milan, Italy; 2 Institute for Biomedical Technologies (ITB)-CNR, Segrate, Milan, Italy; 3 DISMA, Università degli Studi di Milano, Milan, Italy; 4 Infections and Cystic Fibrosis Unit, Division of Immunology, Transplantation and Infectious Diseases, San Raffaele Scientific Institute, Milan, Italy; The Scripps Research Institute and Sorrento Therapeutics, Inc., United States of America

## Abstract

We report on specific magneto-capturing followed by Multidimensional Protein Identification Technology (MudPIT) for the analysis of surface-exposed proteins of intact cells of the bacterial opportunistic pathogen *Pseudomonas aeruginosa*. The magneto-separation of cell envelope fragments from the soluble cytoplasmic fraction allowed the MudPIT identification of the captured and neighboring proteins. Remarkably, we identified 63 proteins captured directly by nanoparticles and 67 proteins embedded in the cell envelope fragments. For a high number of proteins, our analysis strongly indicates either surface exposure or localization in an envelope district. The localization of most identified proteins was only predicted or totally unknown. This novel approach greatly improves the sensitivity and specificity of the previous methods, such as surface shaving with proteases that was also tested on *P. aeruginosa*. The magneto-capture procedure is simple, safe, and rapid, and appears to be well-suited for envelope studies in highly pathogenic bacteria.

## Introduction

The bacterial cell envelope is a highly-structured multi-layer that guarantees cell integrity and protection from environmental adversities while supporting in-out passage of nutrients and wastes [1]. In Gram-negative bacteria, the surface layer consists of an outer membrane (OM) of phospholipids and lipopolysaccharides. OM delimits a periplasmic space that includes a peptidoglycan layer. Finally, the lower layer is a phospholipid inner membrane (IM) that is in contact with the cytoplasm. Proteins play fundamental roles in envelope functions and they may localize in a specific envelope layer. For example, surface proteins localize in OM and participate in interactions with the environment, sensing the chemical and physical conditions of the surroundings and transmitting appropriate signals to the cytoplasm [2]. These functions include adhesion to and, when possible, invasion of physical and biological supports (e.g. host cells for pathogens), as well as the transport of nutrient molecules. Given these essential roles in bacterial life and pathogenicity, identification and characterization of envelope proteins may lead to novel antibacterial targets. Moreover, since surface proteins face the host immune system, they could be the basic elements of effective vaccines [3].

Proteomics studies on the bacterial cell envelope are mainly performed through the separation of the different envelope layers (for Gram-negative bacteria: OM, IM and periplasmic fractions, respectively) from the cytoplasmic fraction [4]. Localization prediction algorithms [5] can also support studies on the envelope proteome. Nonetheless, *in silico* methods are usually designed for precision over recall and, as a result, the localization(s) of some protein classes is not easily predicted [5]. More recently, proteomic studies specifically targeting surface-exposed proteins have used proteases to “shave” intact bacterial cells. This approach has the valuable outcome of directly identifying cell surface-exposed domains of envelope proteins. It has been successfully used for Gram-positive bacteria [6]–[8]. Protease shaving of Gram-negative bacteria surface appears less straightforward, as Gram-negative bacteria are more sensitive than Gram-positive to protease treatment. Proteolysis may impact OM integrity and the ensuing cells lysis can result in massive contamination of the shaved peptides by cytoplasmic proteins [3].

*Pseudomonas aeruginosa* is a highly adaptable Gram-negative bacterium which thrives in a broad range of ecological niches and can infect multiple hosts as diverse as plants, nematodes and mammals. In humans, it is an important opportunistic pathogen [9]. *P. aeruginosa* is a major concern to medical practitioners who increasingly face extremely-drug resistant strains [10], [11]. The development of alternative effective antibacterials and vaccines against *P. aeruginosa* can benefit from the sensitive profiling of cell envelope/surface for the identification of therapeutic candidate targets.

**Figure 1:**
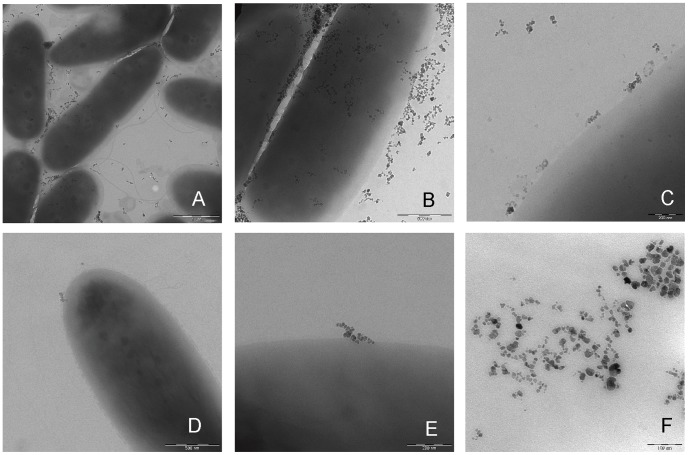
Electron microscopy analysis of *P. aeruginosa* cells treated with NPs. *P. aeruginosa* cells were incubated for 5 min with activated NPs. Tris-HCl was then added to inactivate NPs. A sample (S1) of NPs/cells mix was placed on a Formvar-coated Cu grid for Electron Microscopy (EM) analysis. (A), (B) and (C) Three EM images with increasing magnification of S1 *P. aeruginosa* cells, respectively. Free NPs and NPs interacting with cells can be observed. To remove NPs that had not interacted covalently with cells, NPs/cells mix was filtered through a 0.22 µm filter. Bacterial cells retained by the filter were resuspended in distilled water and a sample of them (S2) was placed on a Formvar-coated Cu grid for EM analysis. (D) and (E) Two EM images with increasing magnification of S2 *P. aeruginosa* cells, respectively. In S2, it can be noted that filtration eliminated most of the NPs interacting with cells, leaving a minority localizing at the cell surface. These experiments showed that cells do not internalize NPs. (F) Activated NPs before addition to cells.

In this work we aimed to develop alternative tools for the proteomic analysis of the bacterial cell envelope. Using *P. aeruginosa* as a model organism, we describe a novel method based on nanoparticles for the magneto-separation of cell envelope fragments from the soluble cytoplasmic fraction. These were used for the MudPIT (Multidimensional Protein Identification Technology) identification of the captured and the neighboring proteins. We identified 63 proteins captured directly by the nanoparticles and 67 proteins embedded in the cell envelope fragments. For surface protein detection, our approach greatly improves the sensitivity and specificity of previous methods such as surface shaving with proteases.

**Figure 2:**
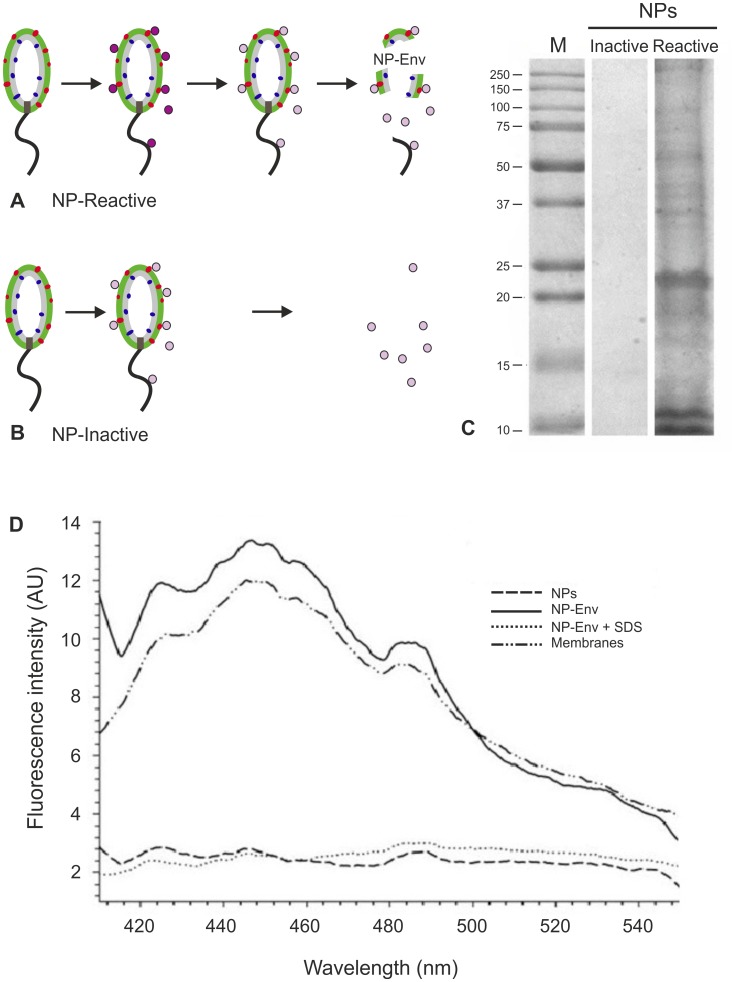
Validation of NPs as magneto-capture tools of envelope structures. (A) Scheme of treatment of *P. aeruginosa* intact cells with activated NPs. Before treatment with activated NPs (dark purple circles), cells were washed by the culture medium to remove extracellular proteins. After treatment for 5 min, NPs were inactivated (pink circles) and cells disrupted. NPs were magnetically recovered and washed thoroughly. NPs that interact with cell surface can establish covalent bonds with free -NH_2_ moieties (e.g. those of lysine of exposed proteins, red dots) and, upon cell lysis, envelope fragments that stick to NPs (NP-Env) can be magneto-captured. (B) Scheme of treatment with inactive NPs. Before treatment with inactive NPs (pink circles), cells were washed by the culture medium to remove extracellular proteins. Upon treatment for 5 min, cells were disrupted. NPs were magnetically recovered and washed thoroughly. Inactive NPs can interact with cell surface but no covalent bonding occurs and thus envelope fragments are not magneto-captured. (C) Reactive and inactive NPs, that had been used to treat *P. aeruginosa* intact cells as illustrated in (A) and (B), respectively, were loaded onto SDS-PAGE to analyze protein contents. M: protein molecular weight marker. (D) Fluorescence emission spectra (λ_ex_: 390 nm; λ_em_: 400–550 nm) of: unreacted NPs (NPs); NP-Env; NP-Env extensively washed with SDS at 60°C (NP-Env+SDS); total membrane preparation (Membranes). All spectra were taken in the presence of the hydrophobic fluorescent probe 0.1 mM 1-anilinonaphthalene-8-sulfonate, tracking the presence of lipids. Note the overlapping spectra of NP-Env and Membranes.

## Methods

### Bacterial Growth Conditions

*P. aeruginosa* PAO1 [12] was routinely grown in BHI broth (Brain Heart Infusion, Sigma) at 37°C. Surface proteome analyses were performed on PAO1 cells grown overnight, diluted to an A_600_ of 0.05 in BHI broth and re-grown with agitation at 37°C until the culture reached an A_600_ of 0.3 (early exponential phase). PAO1 cells were harvested by centrifugation at 4000*×g* for 15 min at 4°C and washed twice with PBS supplemented with 20% sucrose (TIB buffer).

### Surface Digestion of Intact Bacterial Cells (Trypsin Shaving)

PAO1 cells in TIB buffer were incubated with stirring for 30 min at 37°C with 2.5 U/ml trypsin (Sigma). Cells were removed by centrifugation and supernatants containing the peptides released from cell surface were filtered through 0.22 µm filters. After addition of fresh trypsin (40 µg/ml), digestion of the filtered supernatant was prolonged overnight at 37°C to allow the extensive proteolysis of released peptides required for MudPIT analysis.

**Table 1:** List of envelope-associated proteins covalently bound by NPs at cell surface (NP-CbP).

Gene name and/or PA *locus*	Protein name	Protein family	Function classa,e	Localization confidence^e^(CC^b^, class^c^)	NP-EnP^d^
oprF PA1777	OM porin F OprF	OmpA	1	OM,1 - P,1	+
pal oprL PA0973^f^	Peptidoglycan-associated lipoprotein OprL	OmpA	1	OM,1 - P,1	+
icmP PA4370^f^	Insulin-cleaving metalloproteinase OM protein		1	OM,1	+
oprI PA2853^f^	Major OM lipoprotein OprI		1	OM,1	
oprE PA0291	Anaerobically-induced OM porin OprE		1	OM,1 - P,1	
oprH PA1178	OM protein H1 PhoP/Q		1	OM,1 - P,1	
pilQ PA5040	Fimbrial assembly protein PilQ	GSP D	1	OM,1 - P,1	
pagL PA4661	Lipid A 3-O-deacylase PagL		1	OM,1 - P,1	
fpvA PA2398	Ferripyoverdine receptor	TonB-dependent receptor	1	OM,1 - P,1	
foxA PA2466	Ferrioxamine receptor FoxA	TonB-dependent receptor	1	OM, 1	
PA3988^f^	Putative uncharacterized protein		4	OM,1 - P,1	
lptD imp ostA PA0595	LPS-assembly protein LptD	LptD	2	OM,2 - P,1	
PA1041^f^	Putative OM protein	OmpA	3	OM,2	
PA0641	Putative bacteriophage protein		3	OM,2	
PA1271	Putative tonB-dependent receptor	TonB-dependent receptor	3	OM,2 - P,1	
PA2800	Putative uncharacterized protein		4	OM,2 - P,1	
PA0833^f^	Putative uncharacterized protein	OmpA	4	OM,2 - P,1	
PA1053^f^	Putative uncharacterized protein		4	OM,2	
PA0070^f^	Putative uncharacterized protein		2	P,1	
pilA fimA PA4525	Pilin	N-Me-Phe pilin	1	E,3	+
fliD PA1094	B-type flagellar hook-associated protein 2	FliD	1	F,1 - P,1	
mexE PA2493^f^	RND multidrug efflux protein MexE		1	IM,3	+
pctA PA4309	Chemotactic transducer PctA		1	IM,3	+
PA4431	Putative Ubiquinol-cytochrome c reductase		3	IM,3	+
PA3641	Putative amino acid permease		3	IM,3	
PA4423^f^	Putative uncharacterized protein		4	IM,3 - P,1	
fimV PA3115	Motility protein FimV		1	U,3	+
PA4639^f^	Putative uncharacterized protein		4	U,3	
PA0505	Putative uncharacterized protein		4	U,3	
PA3031^f^	Putative uncharacterized protein		4	U,3	
rpsH PA4249	30S rP S8		2	C,1	+
algP algR3 PA5253	Transcriptional regulatory protein AlgP		1	C,3 - U,3	+
tsf PA3655	Elongation factor EF-Ts		2	C,3 - P,1	+
nusG PA4275	Transcription antitermination protein NusG		2	C,3	+
rplJ PA4272	50S rP L10		2	C,3	+
rpmB PA5316	50S rP L28		2	C,3	+
rpsC PA4257	30S rP S3		2	C,3	+
rpsB PA3656	30S rP S2		2	C,3,P,1	+
rpsP PA3745	30S rP S16		2	C,3	+
rpsL PA4268	30S rP S12		2	C,3	+
rpsK PA4240	30S rP S11		2	C,3	+
rpsD PA4239	30S rP S4		2	C,3	+
rplE PA4251	50S rP L5		2	C,3	+
ftsA PA4408	Cell division protein FtsA		2	C,3	+
dnaJ PA4760	Chaperone protein DnaJ		2	C,3	+
mreB PA4481	Rod shape-determining protein MreB		2	C,3	+
PA4595	Putative ABC transporter	ABC transporter	3	C,3 - P,1	+
amrZ PA3385	Alginate and motility regulator Z AmrZ		1	C,1	
infC PA2743	Translation initiation factor IF-3		2	C,1	
rplM PA4433	50S rP L13		2	C,1	
rpsU PA0579	30S rP S21		2	C,1	
rplR PA4247	50S rP L18		2	C,1	
rplW PA4261	50S rP L23		2	C,1	
rpsN PA4250	30S rP S14		2	C,1	
phaF PA5060	Polyhydroxyalkanoate synthesis protein PhaF		2	C,1	
PA3940	Putative DNA binding protein		3	C,1	
proB PA4565	Glutamate 5-kinase ProB		2	C,2	
rpsQ PA4254	30S rP S17		2	C,3	
rplB PA4260	50S rP L2		2	C,3	
rpmD PA4245	50S rP L30		2	C,3	
rplS PA3742	50S rP L19		2	C,3	
rpmF PA2970	50S rP L32		2	C,3	
rluB PA3179	Putative ribosomal pseudouridine synthase B		4	C,3	

a Class 1: Function experimentally demonstrated in *P. aeruginosa*; Class 2: Function of highly similar gene experimentally demonstrated in another organism; Class 3: Function proposed based on presence of conserved amino acid motif, structural feature or limited sequence similarity to an experimentally studied gene. Class 4: Homologs of previously reported genes of unknown function, or no similarity to any previously reported sequences.

b CC: Cell compartment. OM: outer membrane; P: periplasm; E: extracellular; F: flagellar; IM: inner membrane; U: unknown; C: cytoplasmic.

c Class 1: Subcellular localization experimentally demonstrated in *P. aeruginosa*; Class 2: Subcellular localization of highly similar gene experimentally demonstrated in another organism or to a paralog experimentally demonstrated in the same organism. BLAST expected value of 10e-10 for query within 80–120% of subject length. Class 3: Subcellular localization computationally predicted by PSORT.

d Proteins identified also by trypsin digestion of NP-Env.

e Functional class and localization confidence is indicated according to the annotations in Pseudomonas Genome Database (www.pseudomonas.com) [31].

f Lipoprotein, known or predicted [24].

**Table 2:** List of envelope-associated proteins not directly bound by NPs.

Gene name and/or PA *locus*	Protein name	Protein family	Function classa,d	Localization confidence^d^(CC^b^, class^c^)
PA3262^e^	Peptidyl-prolyl cis-trans isomerase	FKBP-type PPIase	3	OM,2
dacC PA3999	Penicillin-binding protein 5		2	P,2 - IM,2
mexA PA0425^e^	Multidrug resistance protein MexA	Membrane fusion protein	1	IM,1 - OM,1
secD PA3821	Protein translocase subunit SecD	SecD/SecF	2	IM,2
ftsH PA4751	Zinc metalloprotease FtsH	Peptidase M41	2	IM,2
msbA PA4997	Lipid A export protein MsbA	ABC transporter	2	IM,2
PA4461	Putative ABC transporter	ABC transporter	3	IM,2
zipA PA1528	Cell division protein ZipA	ZipA	2	IM,3
ppiD PA1805	Peptidyl-prolyl cis-trans isomerase D		2	IM,3 - P,1
secG PA4747	Protein-export protein SecG	SecG	2	IM,3
rho PA5239	Transcription termination factor Rho		2	IM,3 - P,1
atpF PA5558	ATP synthase subunit b	ATPase	2	IM,3
sdhA PA1583	Succinate dehydrogenase (A subunit)		2	IM,3 - P,1
sdhB PA1584	Succinate dehydrogenase (B subunit)		2	IM,3
lepA le PA0767	Elongation factor EF-4		2	IM,3
typA PA5117	Regulatory protein TypA		2	IM,3 - P,1
pssA PA4693	Phosphatidylserine synthase		2	IM,3
gcd PA2290	Glucose dehydrogenase		2	IM,3
PA2652	Putative chemotaxis transducer		3	IM,3
oxaA PA5568	Putative protein OxaA	OXA1/oxaA	4	IM,3
PA5528	Putative uncharacterized protein		4	IM,3
PA3729	Putative uncharacterized protein		4	IM,3
PA2873	Putative uncharacterized protein		4	IM,3
PA5258	Putative uncharacterized protein		4	IM,3
ccoP1 PA1552	Cytochrome c oxidase subunit		1	U,3
hflK PA4942	Protease subunit HflK		2	U,3
PA0537^e^	Putative uncharacterized protein		4	U,3
PA4441	Putative uncharacterized protein		4	U,3
PA1592^e^	Putative uncharacterized protein		4	U,3
PA5146	Putative uncharacterized protein		4	U,3
PA4961	Putative uncharacterized protein		4	U,3
PA4842	Putative uncharacterized protein		4	U,3
PA0126	Putative uncharacterized protein		4	U,3
alaS PA0903	Alanyl-tRNA synthetase AlaS		2	C,1 - P,1
rpsA PA3162	30S rP S1		2	C,1 - P,1
rpsE PA4246	30S rP S5		2	C,1
aspS PA0963	Aspartyl-tRNA synthetase AspS		2	C,2 - P,1
lon PA1803	Lon protease		2	C,2
aceF aceB PA5016	Dihydrolipoyllysine-residue acetyltransferase		1	C,3 - P,1
rpoD PA0576	RNA polymerase sigma factor RpoD		1	C,3
ccoO1 PA1553	Cytochrome c oxidase		1	C,3
ftsY PA0373	Signal recognition particle receptor FtsY		2	C,3
mqo1 mqoA PA3452	Putative malate:quinone oxidoreductase 1		2	C,3
PA0084	Putative uncharacterized protein		2	C,3
ibpA PA3126	Heat-shock protein IbpA		2	C,3
tig PA1800	Trigger factor (TF)		2	C,3 - P,1
nusA PA4745	N utilization substance protein A NusA		2	C,3 - P,1
infB PA4744	Translation initiation factor IF-2		2	C,3 - P,1
rne PA2976	Ribonuclease E		2	C,3
accA PA3639	Acetyl-coenzyme A carboxylase		2	C,3
atpD PA5554	ATP synthase subunit beta	ATPase	2	C,3 - P,1
atpA PA5556	ATP synthase subunit alpha	ATPase	2	C,3 - P,1
clpX PA1802	Clp protease ClpX		2	C,3
gyrB PA0004	DNA gyrase subunit B		2	C,3 - P,1
secA PA4403	Protein translocase subunit SecA	SecA	2	C,3
clpV1 PA0090	Protein ClpV1		2	C,3
pcnB PA4727	Poly(A) polymerase		2	C,3
atpG PA5555	ATP synthase gamma chain	ATPase	2	C,3
atpC PA5553	ATP synthase epsilon chain	ATPase	2	C,3
rpoA PA4238	RNA polymerase subunit alpha RpoA		2	C,3
PA3019	Putative ABC transporter	ABC transporter	3	C,3
PA1964	Putative ABC transporter	ABC transporter	3	C,3
PA1458	CheA homolog		3	C,3
PA2735	Putative restriction-modification system protein		3	C,3
PA2840	Putative RNA helicase		3	C,3
PA3804	Putative uncharacterized protein		4	C,3 - U,3
PA4438	Putative uncharacterized protein		4	C,3

a Class 1: Function experimentally demonstrated in *P. aeruginosa*; Class 2: Function of highly similar gene experimentally demonstrated in another organism; Class 3: Function proposed based on presence of conserved amino acid motif, structural feature or limited sequence similarity to an experimentally studied gene. Class 4: Homologs of previously reported genes of unknown function, or no similarity to any previously reported sequences.

b CC: Cell compartment. OM: outer membrane; P: periplasm; E: extracellular; F: flagellar; IM: inner membrane; U: unknown; C: cytoplasmic.

c Class 1: Subcellular localization experimentally demonstrated in *P. aeruginosa*; Class 2: Subcellular localization of highly similar gene experimentally demonstrated in another organism or to a paralog experimentally demonstrated in the same organism. BLAST expected value of 10e-10 for query within 80–120% of subject length. Class 3: Subcellular localization computationally predicted by PSORT.

d Functional class and localization confidence is indicated according to the annotations in Pseudomonas Genome Database (www.pseudomonas.com) [31].

e Lipoprotein, known or predicted [24].

### Preparation and Activation of Magnetic Nanoparticles

Iron oxide magnetic nanoparticles (NPs) of diameter 70–90 nm were synthesized by chemical co-precipitation from an aqueous solution of Fe^3+^, Mn^2+^, and Zn^2+^ chloride, and coated with carboxymethyl dextran [13]. Following extensive washing, the NPs coating was activated [14] by incubation for 30 min in 50 mM MES buffer pH 6.0, containing 100 mM carbodiimide (EDC) and 100 mM N-hydroxysuccinimide. Activated NPs were separated by centrifugation and used immediately.

### Binding of Envelope Proteins to Nanoparticles

PAO1 cells in TIB buffer were incubated for 5 min at 37°C under stirring in the presence of activated NPs (0.5 mg/ml). Reactive groups on the NPs surface were then blocked with Tris-HCl (pH 7.4) added to a final concentration of 200 mM. After cell disruption in a French press device, NPs were recovered through a permanent magnet for 1 h at 4°C, and washed four times - twice with water and twice with 1 M NaCl - to remove non-specifically bound material. In control experiments, NPs were inactivated by treatment with Tris-HCl buffer prior to being added to cells.

### Electron Microscopy

PAO1 cells in TIB buffer were diluted to 10^5^–10^6^ CFU/ml and incubated for 5 min with activated NPs (0.5 mg/ml). Tris-HCl (200 mM, pH 8.0) was then added to inactivate NPs. 3 µl of NPs/cells mix were placed on a Formvar-coated Cu grid. To remove unbound NPs, the NPs/cells mix was filtered through a 0.22 µm filter. Bacterial cells retained by the filter were resuspended in 1 ml of distilled water and 3 µl of the cell suspension was placed on a Formvar-coated Cu grid. The specimens were examined with a LEO912 AB energy-filtering transmission electron microscope (EFTEM) (Carl Zeiss, Oberkochen, Germany) operating at 80 kV. Digital images were recorded with a ProScan 1K Slow-Scan charge-coupled device (CCD) camera (Proscan, Scheuring, Germany). Images were processed with Adobe Photoshop for contrast adjustment (Image-adjustments-autocontrast).

### Identification of NP-bound Proteins

To remove non-covalently bound proteins and fragments of the cell envelope, washed NPs from the binding experiments (NP-Env) were incubated for 1 hr at 60°C in the presence of 1% SDS. The nature and the amount of the released proteins were assessed by SDS-PAGE. The proteins that remained covalently bound to the SDS-treated NPs (NP-CbP) were then digested with 40 µg/ml trypsin to break them into fragments suitable for MudPIT identification. In a different set of experiments, washed NP-Env were directly treated with 40 µg/ml trypsin overnight at 37°C with stirring.

### MudPIT Analysis

Trypsin-digested samples resulting from the approaches described above were desalted on PepClean C-18 Spin Columns (PIERCE Biotechnology Inc., Rockford, IL, USA) and analyzed by means of two-dimensional micro liquid chromatography coupled with an ion trap mass spectrometer (2DC-MS/MS, also referred to as Multidimensional Protein Identification Technology (MudPIT), using the ProteomeX configuration (Thermo Fisher, San Josè, CA, USA). Briefly, an autosampler (MicroAS Thermo Fisher Scientific, San Josè, CA, USA) was used to load 10 µl of the digested peptide mixtures into a strong cation exchange column (Biobasic-SCX, 0.32 i.d.×100 mm, 5 µm, Thermo Scientific, Bellefonte, PA, USA) that was eluted stepwise by increasing the ammonium chloride molarity (0, 20, 40, 80, 120, 200, 400, 700 mM). Eluates from each salt step were captured on-line onto peptide traps (Zorbax 300SB-C18, 5×0.3 mm, 5 µm, Agilent Technologies, Palo Alto, CA, USA) for concentration and desalting prior to being loaded into a reverse-phase C18 column (Biobasic-18, 0.180 i.d.×100 mm, 5 µm, Thermo Scientific, Bellefonte, PA, USA) and separated with an acetonitrile gradient (eluent A, 0.1% formic acid in water; eluent B, 0.1% formic acid in acetonitrile). The gradient profile was 5 to 10% eluent B in 5 minutes, 10 to 40% B in 40 minutes, 40 to 80% B in 8 minutes, and 80 to 95% in 3 minutes. Flow-rate was 1 µl/min. The eluted peptides were analyzed directly with an ion trap mass spectrometer (LCQ Deca XP Plus, Thermo Fischer, San Josè, CA, USA), equipped with a nano electrospray ionization source. The spray capillary voltage was set at 1.7 kV, while the ion transfer capillary temperature was maintained at 185°C. Full mass spectra were acquired in positive mode and over a 400–2000 m/z range, followed by four MS/MS events sequentially generated in a data-dependent manner on the first, second, third and fourth most intense ions selected from the full MS spectrum (collision energy 35%), using dynamic exclusion for MS/MS analysis.

The experimental mass spectra produced by MudPIT analyses were correlated to *in silico* peptide sequences of a non-redundant *P. aeruginosa* protein database (5753 entries) retrieved from NCBI (http://www.ncbi.nlm.nih.gov/). Data processing of raw spectra was performed by the Bioworks 3.3.1 software (University of Washington, licensed to Thermo Fisher Scientific), based on the SEQUEST algorithm [15], in combination with a cluster PC. No enzyme specificity was assumed and parent and fragment ion tolerance was set to 2 and 1 atomic mass units (amu), respectively. Peptide and protein assignment were obtained by applying filtering criteria according to established guidelines [16]. Specifically, matching between spectra was retained only if they had a minimum X_corr_ of 1.5 for +1, 2.0 for +2 and 2.5 for +3 charge state. The thresholds of peptide/protein probability were determined to ≤10^−3^ and a protein consensus score ≥10. Only a subgroup of high-confidence proteins, identified with two or more spectral counts or by high identification frequency (≥40%) was considered. The percentage of false positives identification was estimated by processing the raw mass spectra with the reverse database of *P. aeruginosa* as reported previously [17]. The procedure revealed a false positives rate <5% (data not shown).

### Statistical Comparison of MudPIT Data

Lists of MudPIT-identified proteins resulting from shaving of intact cells or from NP-Env experiments were compared to lists of MudPIT-identified proteins of “shedding” controls as follows. The ensuing data were handled and semi-quantitatively compared by applying DAve (Differential Average) and DCI (Differential Confidence Index) algorithms as inserted in the MAProMa software [18]. In addition, a final validation of the identified differences was performed using the statistical G-test to better assess the accuracy of the selection criteria [19]. Briefly, G was calculated according to equation (1) [20]:
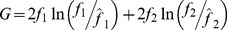
(1)where f_1_, f_2_ are the normalized spectral count for the protein in sample 1 and sample 2, and 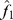, 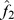 are expected SpC for the protein in sample 1 and sample 2. Assuming that the protein is equally expressed, then 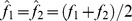. Semi-quantitative differences were retained with a significance accepted at *P*<0.05.

## Results and Discussion

For the capture of *P. aeruginosa* surface-exposed proteins, carboxymethyl-dextran coated magnetic nanoparticles (NPs) were activated allowing them to establish covalent bonds with exposed lysine groups in proteins [21]. Given their chemical composition, size (average diameter = 70–90 nm), and negative charge, these NPs were expected to be atoxic for bacterial cells, as they are for eukaryotic cells [21]. Viable counts of bacterial cells incubated with and without NPs indicated no lethality (Figure S1B). Electron microscopy evidence (Figure 1) also indicated that NPs were unable to pass through cell envelope and distributed over the cell surface.

As shown in Figure 2A, activated NPs were incubated with bacterial cells and, subsequently, their reactive groups were inactivated. Cells were disrupted, and NPs were magnetically separated from cell extracts, washed, and tested for protein and cell-membrane content. The SDS-PAGE tracings in Figure 2C show that only activated NPs collected proteins from cells whereas no proteins were detected in the controls (Figure 2B) carried out with NPs inactivated prior to exposure to cells. MudPIT analysis of controls further indicated that protein binding activity of inactivated NPs was negligible. Membranes were also associated with proteins collected by activated NPs, as shown by the differential binding of the hydrophobic fluorescent probe, 1-anilino-8-naphtalen sulfonate [22] (Figure 2D) to NP-bound structures following treatment at 60°C in the presence of detergents. Therefore, NPs could specifically fish cell envelope structures (NP-Env, Figure 2A).

For envelope proteomic analysis, NP-Env were subjected to two different treatments (Figure S2A): a) they were treated with trypsin in the absence of other treatments to digest envelope-anchored proteins. For membrane proteins, this procedure was expected to shave protruding domains. Tryptic peptides released from NP-Env were identified by MudPIT analysis. Envelope-associated proteins identified by this treatment will be referred to as NP-EnP; b) alternatively, heat and detergents were used to remove all envelope material from NP-Env except the proteins that were covalently bound to NPs. The NPs-bound proteins were also identified by MudPIT analysis. This treatment aimed to identify proteins with outer-exposed domains that were covalently bound by NPs at intact cell surface. These proteins will be referred to as NP-CbP.

The activity of NPs to capture proteins shed spontaneously from intact cells [7] and proteins released by spontaneous cell lysis was evaluated through control experiments (NP-Shed). In these controls, the cells were incubated without NPs, removed by tandem centrifugation-filtration, and the resulting supernatants were incubated with activated NPs; in this way, NPs could only react with released proteins (Figure S2B). NPs were then inactivated, and the proteins covalently bound to NPs were identified by MudPIT analysis. Finally, we listed the proteins genuinely digested from NP-Env (NP-EnP) or covalently bound by NPs at the cell surface (NP-CbP) by statistical comparison of the MudPIT results of NP-Env treatments a) and b), respectively, with those of NP-Shed control. As a result, 92 and 63 proteins were identified following treatments a) and b), respectively (see Supplementary Tables S1 and S2 for raw data of identification).

Table 1 lists the 63 proteins identified as NP-CbP with surface-exposed domains. As expected, some of them were also identified as NP-EnP. Significantly, 11 NP-CbP have experimentally demonstrated subcellular localization in the outer membrane while, for 7 NP-CbP, a localization in the outer membrane was experimentally demonstrated for orthologous and paralogous proteins. A periplasmic localization was also observed for many of these proteins in *P. aeruginosa* [23]. Moreover, the number of lipoproteins, known or predicted in *P. aeruginosa* [24], is remarkable among NP-CbP hits listed in Table 1.

Not surprisingly, pilin and FliD (a flagellar hook-associated protein) were detected as NP-CbP. Instead, an interesting result was that MexE, PctA, PA4431, PA3641 and PA4423 were predicted to localize in IM, but were bound directly by NPs. This may suggest that these proteins localize in an envelope district wider than IM and extending outward. The notion of residence in a wide district may also be true for a number of proteins of Table 1 having predicted or experimentally determined cytoplasmic localization. Multiple localizations are not unusual for a protein. For example, “classical” cytoplasmic proteins, such as ribosomal proteins, along with other anchorless proteins, have been observed on bacterial surfaces in many studies [6], [7], [25], [26]. However, their function at the envelope level remains unclear. Finally, 4 proteins (FimV, PA4639, PA0505, and PA3031), detected as NP-CbP, had an unknown localization according to PSORTb 3.0 [5]. Our results would strongly suggest that they have at least surface localization and this could overcome the lack of information on proteins whose localization is not easily predicted.

Table 2 lists 67 envelope-associated proteins not directly bound by NPs and exclusively identified as NP-EnP. It differs to Table 1 in that only two proteins, PA3262 and MexA, have predicted or experimentally determined OM localization, respectively. They might not be captured by NPs due to the lack of surface-exposed domains. Otherwise, as expected from trypsin treatment of NP-Env, the number of experimentally demonstrated (MexA; [27]) or predicted proteins that localize in IM is significantly higher than NP-CbP shown in Table 1 (23 *vs* 5, respectively). Remarkably, trypsin treatment of NP-Env can have shaved protein domains emerging from IM, both from cytoplasmic and periplasmic side. Mapping of the resulting peptides along primary sequences can provide experimental data to correlate with the protein topology predicted by suitable software (e.g. TMHMM Server v.2.0; [28]). Nine NP-EnP listed in Table 2 had an unknown localization according to PSORTb 3.0 [5]. Our results strongly suggest that they belong to the envelope district at least. Finally, as in the case of NP-CbP, several proteins have either predicted or experimentally determined cytoplasmic localization. It should be mentioned that our envelope analysis detected as NP-EnP the α, β, γ, ε and b subunits of ATP synthase (Table 2), belonging to the cytoplasmic portion of the IM-associated ATP synthase F_0_F_1_ complex [29]. Consequently, the NP-Env trypsin treatment appears to be well-suited for analysis/identification of proteins that play a cytoplasmic function when assembled in membrane complexes. The same may be true for proteins that assemble on the IM periplasmic side.

In the list of NP-EnP in Table 2, 34 proteins have localization confidence in cytoplasm. Three proteins, AlaS and ribosomal proteins S1 and S5 have an experimentally determined localization in cytoplasm [5], [30]. AlaS and S1 have been also detected in periplasm [23]. The remaining 31 proteins have predicted cytoplasmic localization, 8 of them have been experimentally determined in periplasm [23]. Therefore, the role of these proteins could not be restricted exclusively to the cytoplasm but may act also in the envelope in the same way as AlaS and S1 (see above).

We aimed to compare the NP-based envelope analysis described above with a cell surface scanning performed by trypsin shaving of intact bacterial cells. This latter approach has been successfully used for Gram-positive bacteria [6]–[8]. However, shaving of Gram-negative bacteria surface appears less straightforward. Apparently, Gram-negative bacteria are more sensitive to protease treatment. Proteolysis may impact OM integrity so that the ensuing cells lysis results in massive contamination of the shaved peptides by cytoplasmic proteins [3]. We performed trypsin shaving of intact *P. aeruginosa* cells according to previously described protocols [7] with the experimental scheme depicted in Figure S3A. To minimize the deleterious effects of protease treatment on cell integrity, trypsin concentration and incubation times were adjusted to have less than 2% of cell mortality (evaluated by viable cells count, Figure S1A). During incubation with trypsin, surface-proteins can be shaved, and exposed peptide released (Figure S3A III). However, both surface and non-surface proteins can be shed spontaneously from intact cells [7] and are digested by trypsin once in solution outside the cell. The tryptic peptides deriving from the shed proteins can thus contaminate the pool of those genuinely shaved from surface-exposed proteins, making it difficult to evaluate the outcome of the assay. Spontaneous cell lysis can be another obvious source of contamination. To perform a “shedding” control, cells were thus incubated without trypsin to monitor protein release in the incubation buffer (Figure S3B III). In either case, the shaved peptides and released proteins were collected - after cell removal by tandem centrifugation-filtration - and identified through MudPIT (Figure S3A and B IV-VI). Finally, we generated a list of proteins genuinely shaved by trypsin through statistical comparison of the MudPIT results of cell shaving with those of the “shedding” control. This approach identified a total of 47 proteins (Table S3). Proteinase K was also considered as a shaving tool, but preliminary assays indicated that even mild proteinase K treatment elicited high cell mortality, supposedly by cell lysis. Therefore, we did not proceed further with MudPIT analyses of products of proteinase K treatment. In Table S4, the proteome dataset resulting from NP-EnP and NP-CbP MudPIT analysis (Tables 1 and 2) is compared with those from shaving on intact cells (Table S3).

This comparison makes it evident that NP capture may become a very valuable tool for the study of bacterial surface. Shaving whole cells with proteases – with shedding studies as controls - has the advantage of direct identification of the surface exposed protein domains [7], [8]. However, this approach can have several serious limitations, including: i) underestimation of surface proteins, with the presence of accessible protease-sensitive sites/sequences a necessary prerequisite; ii) cell disruption by the protease treatment, releasing contaminant proteins; iii) limited use with Gram-negative bacteria which appear to be more sensitive to protease treatment than Gram-positive bacteria; iv) partial surface digestion allowing the sole detection of abundant surface proteins, due to the mild trypsin treatment required to preserve cell integrity. On the contrary, NPs docking on cell surface (Figure 1) did not induce cell lysis (Figure S1B). Unlike trypsin shaving on intact cells, the NPs protocol could identify as NP-CbP a relevant number of proteins with high localization confidence in OM (1 *vs* 19, respectively; Table S4). NPs surface interaction and covalent capture appears therefore to be a more sensitive tool than protease shaving for scanning and identifying cell surface exposed proteins. Furthermore, since contamination from trypsin-induced cell lysis is impossible to control, the mildness of NPs-based treatment increases reliability of “at the surface” identification. Another point of reliability is the control of protein shedding and release by spontaneous cell lysis (Figure S2B). Most proteins identified as NP-CbP, were not detected in control experiments, whereas others – although detected in controls - were significantly enriched in surface capture experiments (Figure S2A). Furthermore, NPs allow also the identification and study of non-exposed envelope proteins neighboring the bound ones on NP-Env. By trypsin treatment of NP-Env, we identified many NP-EnP, in a pattern that only partially overlapped with that of NP-CbP. Subfractionation of envelope material in NP-Env is expected to allow identification of a larger set of envelope proteins. Compared to previous methods based on separation of the different envelope layers [4], envelope fragments tethered to magnetic NPs can be better separated and washed from cytoplasmic fraction.

Consequently, characterizing proteins - covalently and non-covalently bound to NPs - could provide a much clearer and complete picture of the envelope system than the one offered by the other “classical” methods. The bacterial envelope proteome is finely regulated by environmental cues. In this work, to develop the method we used *P. aeruginosa* cells grown in a standard rich medium. However, the ease of nano-magnetic-capture makes it scalable to comparative analysis of envelope proteins under different growth conditions, (e.g. exponential *vs* stationary phase; rich *vs* minimal or oligo elements-limited media, etc.). Such approaches are expected to identify differentially regulated groups of envelope proteins. Moreover, the complexity of the classical approaches has to some extent hindered their application with highly pathogenic bacteria. The simplicity of nano-magnetic-capture, associated with the use of non-aerosolizing cell breakage methods, makes NPs well-suited for envelope studies in such risky bacteria.

## Supporting Information

Figure S1 **Effects of trypsin shaving and NPs treatment on** ***P. aeruginosa*** **cell viability.** (A) Increasing concentrations of trypsin were used to treat *P. aeruginosa* cells in TIB buffer for 30 min at 37°C. At the end of treatment, viable counts were determined on BHI agar plates and reported as percentage of viable cells compared to samples that were not treated with trypsin. (B) *P. aeruginosa* cells in TIB buffer were treated with 1 mg/ml activated NPs for 5 min. At the end of treatment, viable counts were determined on BHI agar plates and compared with those of untreated cells.
(TIF)

Figure S2 **Analysis of cell envelope proteome by magneto-capture of surface-exposed proteins.** (A) Scheme of intact cells treatment with reactive NPs. *P. aeruginosa* cells (dark green: outer membrane; light green: inner membrane; red shapes: surface exposed proteins; black shapes: inner membrane proteins) were grown in BHI medium to the mid-exponential phase (I), washed to remove extracellular proteins (blue shapes) (II) and incubated with reactive NPs (dark purple circles) for 5 min (III). NPs were inactivated (pink circles) (IV) and cells disrupted (V). NPs were magnetically recovered and washed thoroughly. Magneto-captured envelope fragments (NP-Env) were digested with trypsin (yellow shapes) (40 µg/ml) for 16 h at 37°C (VI). To identify proteins digested by trypsin (NP-EnP), NPs were magnetically removed and short tryptic peptides in the supernatant were analyzed by MudPIT (VII). Alternatively, NP-Env was treated with heat SDS for 1 hr to remove all envelope material except the proteins that were covalently bound to NPs (NP-CbP) (VI’). NP-CbP were extensively washed to remove SDS and shaved with trypsin (yellow shapes) (40 µg/ml) for 16 h at 37°C (VI’’). NPs were magnetically removed and short tryptic peptides in the supernatant were analyzed by MudPIT (VII). (B) Control scheme of protein shedding (NP-Shed in the main text) in the treatment of intact cells with reactive NPs. *P. aeruginosa* cells, as in (A), were grown in BHI medium until mid-exponential phase (I), washed to remove extracellular proteins (blue shapes) (II) and incubated without NPs for 5 min at 37°C (III). Cells were removed by tandem centrifugation-filtration and reactive NPs were added to the supernatant containing the shed proteins (orange shapes) and incubated for 5 min (IV). NPs were inactivated (pink circles) (V) and the shed proteins bound to NPs were treated with trypsin (yellow shapes) (40 µg/ml) for 16 h at 37°C (VI). NPs were magnetically removed and short tryptic peptides in the supernatant were analyzed by MudPIT (VII).
(TIF)

Figure S3 **Scheme of the surface shaving of intact cells.** (A) Trypsin treatment of intact cells. *P. aeruginosa* cells (dark green: outer membrane; light green: inner membrane; red shapes: surface exposed proteins; black shapes: inner membrane proteins) were grown in BHI medium until mid-exponential phase (I), washed to remove extracellular proteins (blue shapes) (II) and incubated with trypsin (yellow shapes) for 30 min at 37°C (III). Cells were removed by tandem centrifugation-filtration (IV) and the supernatant proteins (orange shapes: shed proteins; red segments: shaved peptides) were extensively digested with fresh trypsin for 16 h (V) to obtain short tryptic peptides (VI) suited to MudPIT analysis. (B) Incubation of intact cells without trypsin as a control of protein shedding. As in (A), *P. aeruginosa* cells were grown in BHI medium to the mid-exponential phase (I), washed to remove extracellular proteins (blue shapes) (II) and incubated without trypsin for 30 min at 37°C (III). Cells were removed by tandem centrifugation-filtration (IV) and shed proteins (orange shapes) were extensively digested with fresh trypsin for 18 h (V) to obtain short tryptic peptides (VI) suited to MudPIT analysis.
(TIF)

Table S1 **List of proteins (NP-EnP) identified by trypsin treatment of NP-Env and considered “shaved” because of the corresponding average Spectral Count (SpC) that resulted significantly higher by G-test (P>95%) than the SpC determined with the “shedding” control NP-Shed. SpC was calculated from the results of 4 MudPIT analyses.**
(PDF)

Table S2 **List of proteins (NP-CbP) identified by the denaturant treatment of NP-Env and considered bound by NPs at the cell surface because of the corresponding average Spectral Count (SpC) that resulted significantly higher by G-test (P>95%) than the SpC determined with the “shedding” control NP-Shed. SpC was calculated from the results of 4 MudPIT analyses.**
(PDF)

Table S3 **List of proteins identified by trypsin treatment of intact cells and considered “shaved” because of the corresponding average Spectral Count (SpC) that resulted significantly higher by G-test (P>95%) than the SpC determined in the absence of a trypsin treatment (“shedding” control). SpC was calculated from the results of 4 MudPIT analyses.**
(PDF)

Table S4 **Summary list of proteins that: i) were found following trypsin shaving of intact cells (Sh), ii) remained covalently bound to NPs after treatment of NP-Env with denaturants (NP-CbP) and iii) were identified after trypsin digestion of NP-Env (NP-EnP).**
(PDF)
