# Supplementary figures and images for: Analysis of Pseudomonas aeruginosa Cell Envelope Proteome by Capture of Surface-Exposed Proteins on Activated Magnetic Nanoparticles
Source: PLoS One. 2012 Nov 30;7(11):e51062. doi: 10.1371/journal.pone.0051062 (PMC3511353; doi:10.1371/journal.pone.0051062)

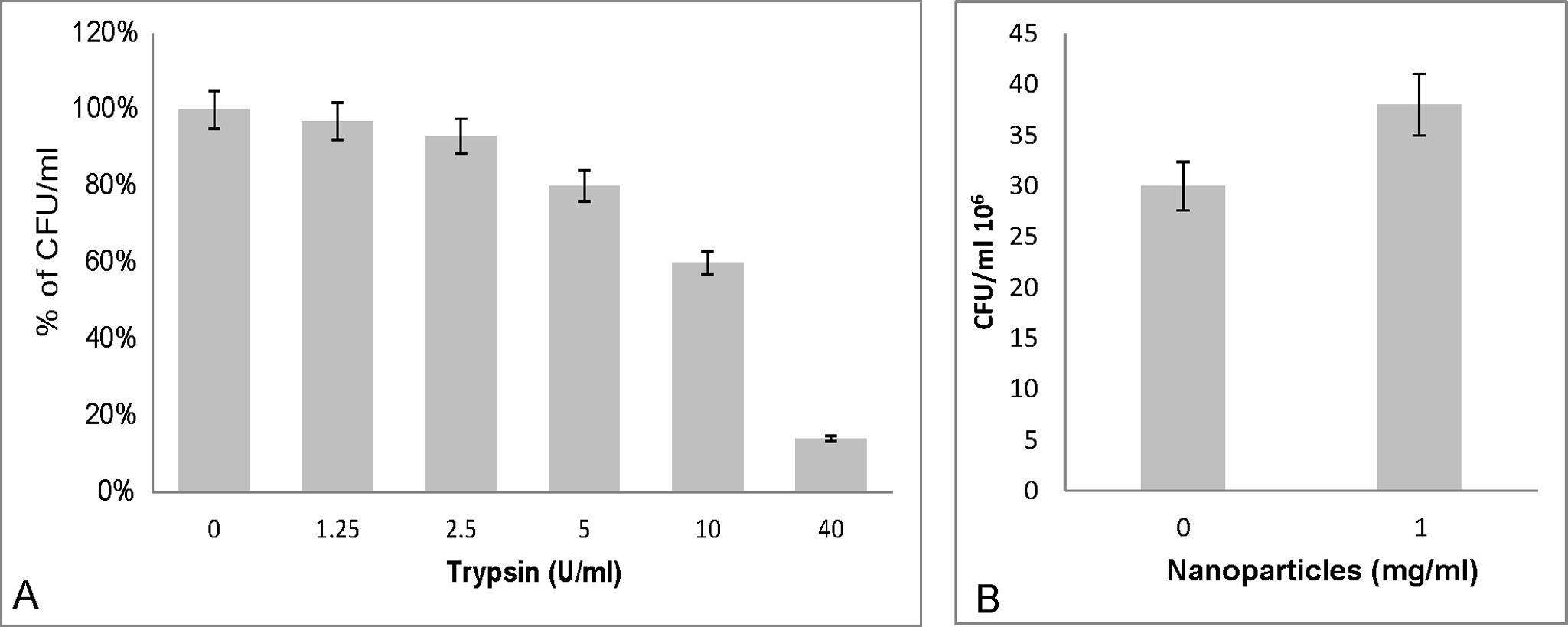

Supplement: Figure S1 — Effects of trypsin shaving and NPs treatment on P. aeruginosa cell viability. (A) Increasing concentrations of trypsin were used to treat P. aeruginosa cells in TIB buffer for 30 min at 37°C. At the end of treatment, viable counts were determined on BHI agar plates and reported as percentage of viable cells compared to samples that were not treated with trypsin. (B) P. aeruginosa cells in TIB buffer were treated with 1 mg/ml activated NPs for 5 min. At the end of treatment, viable counts were determined on BHI agar plates and compared with those of untreated cells. (TIF) [file pone.0051062.s001.tif]

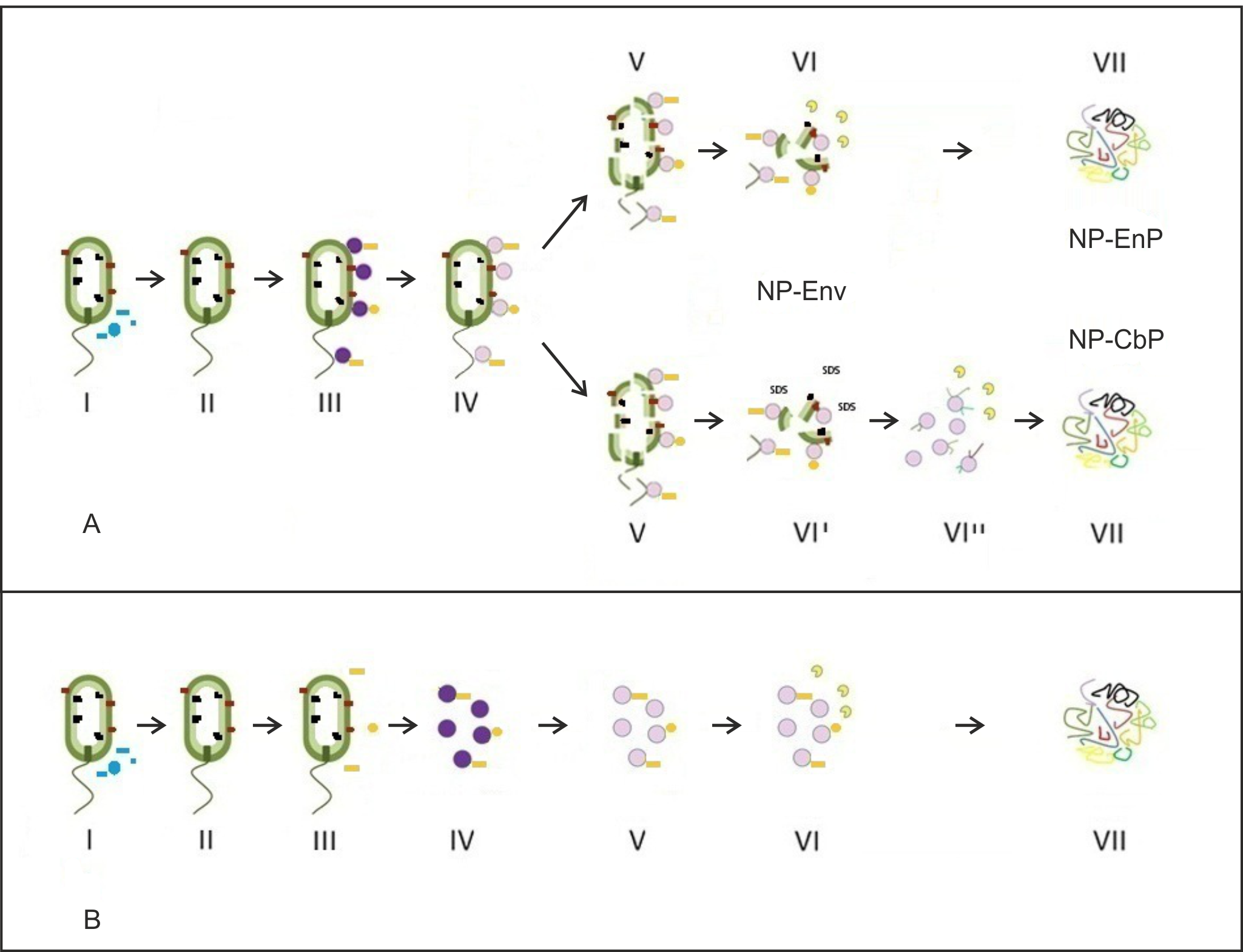

Supplement: Figure S2 — Analysis of cell envelope proteome by magneto-capture of surface-exposed proteins. (A) Scheme of intact cells treatment with reactive NPs. P. aeruginosa cells (dark green: outer membrane; light green: inner membrane; red shapes: surface exposed proteins; black shapes: inner membrane proteins) were grown in BHI medium to the mid-exponential phase (I), washed to remove extracellular proteins (blue shapes) (II) and incubated with reactive NPs (dark purple circles) for 5 min (III). NPs were inactivated (pink circles) (IV) and cells disrupted (V). NPs were magnetically recovered and washed thoroughly. Magneto-captured envelope fragments (NP-Env) were digested with trypsin (yellow shapes) (40 µg/ml) for 16 h at 37°C (VI). To identify proteins digested by trypsin (NP-EnP), NPs were magnetically removed and short tryptic peptides in the supernatant were analyzed by MudPIT (VII). Alternatively, NP-Env was treated with heat SDS for 1 hr to remove all envelope material except the proteins that were covalently bound to NPs (NP-CbP) (VI’). NP-CbP were extensively washed to remove SDS and shaved with trypsin (yellow shapes) (40 µg/ml) for 16 h at 37°C (VI’’). NPs were magnetically removed and short tryptic peptides in the supernatant were analyzed by MudPIT (VII). (B) Control scheme of protein shedding (NP-Shed in the main text) in the treatment of intact cells with reactive NPs. P. aeruginosa cells, as in (A), were grown in BHI medium until mid-exponential phase (I), washed to remove extracellular proteins (blue shapes) (II) and incubated without NPs for 5 min at 37°C (III). Cells were removed by tandem centrifugation-filtration and reactive NPs were added to the supernatant containing the shed proteins (orange shapes) and incubated for 5 min (IV). NPs were inactivated (pink circles) (V) and the shed proteins bound to NPs were treated with trypsin (yellow shapes) (40 µg/ml) for 16 h at 37°C (VI). NPs were magnetically removed and short tryptic peptides in the supernatant were an [file pone.0051062.s002.tif]

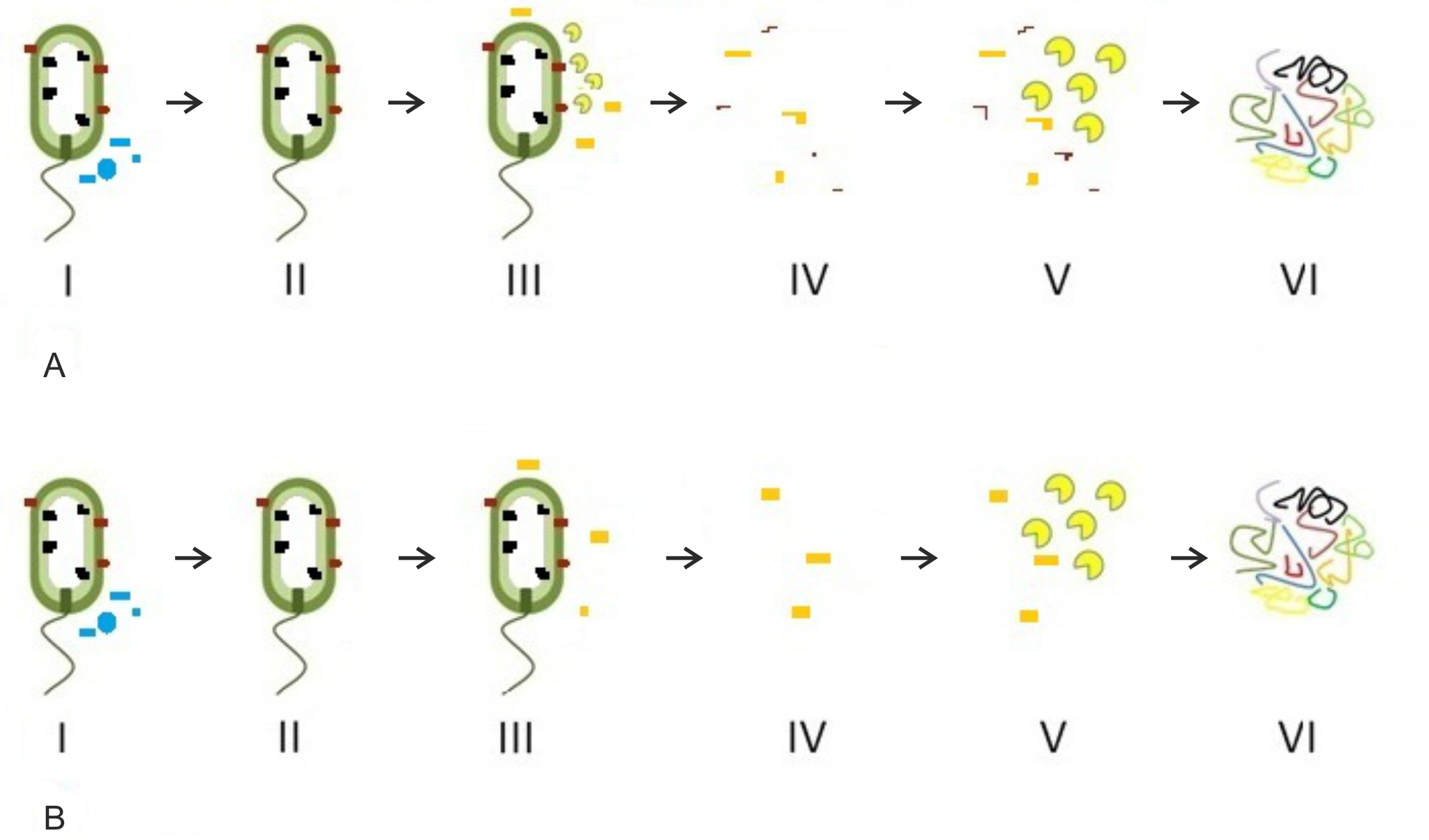

Supplement: Figure S3 — Scheme of the surface shaving of intact cells. (A) Trypsin treatment of intact cells. P. aeruginosa cells (dark green: outer membrane; light green: inner membrane; red shapes: surface exposed proteins; black shapes: inner membrane proteins) were grown in BHI medium until mid-exponential phase (I), washed to remove extracellular proteins (blue shapes) (II) and incubated with trypsin (yellow shapes) for 30 min at 37°C (III). Cells were removed by tandem centrifugation-filtration (IV) and the supernatant proteins (orange shapes: shed proteins; red segments: shaved peptides) were extensively digested with fresh trypsin for 16 h (V) to obtain short tryptic peptides (VI) suited to MudPIT analysis. (B) Incubation of intact cells without trypsin as a control of protein shedding. As in (A), P. aeruginosa cells were grown in BHI medium to the mid-exponential phase (I), washed to remove extracellular proteins (blue shapes) (II) and incubated without trypsin for 30 min at 37°C (III). Cells were removed by tandem centrifugation-filtration (IV) and shed proteins (orange shapes) were extensively digested with fresh trypsin for 18 h (V) to obtain short tryptic peptides (VI) suited to MudPIT analysis. (TIF) [file pone.0051062.s003.tif]
