# Supplementary material for: Analysis of Pseudomonas aeruginosa Cell Envelope Proteome by Capture of Surface-Exposed Proteins on Activated Magnetic Nanoparticles
Source: PLoS One. 2012 Nov 30;7(11):e51062. doi: 10.1371/journal.pone.0051062 (PMC3511353; doi:10.1371/journal.pone.0051062)
Supplement: Table S1 — List of proteins (NP-EnP) identified by trypsin treatment of NP-Env and considered “shaved” because of the corresponding average Spectral Count (SpC) that resulted significantly higher by G-test (P>95%) than the SpC determined with the “shedding” control NP-Shed. SpC was calculated from the results of 4 MudPIT analyses. (PDF) [file pone.0051062.s004.pdf]

Table S1 List of proteins (NP-EnP) identified by trypsin treatment of NP-Env and considered “shaved” because of the corresponding average Spectral Count (SpC) that resulted significantly higher by G-test (P>95%) than the SpC determined with the “shedding” control NP-Shed[a]. SpC was calculated from the results of 4 MudPIT analyses.

Gene names	Protein names	SpC[b]
pal oprL PA0973	Peptidoglycan-associated lipoprotein OprL	7.2
icmP PA4370	Insulin-cleaving metalloproteinase OM protein	16.5
oprF PA1777	OM porin F OprF	17.5
secD PA3821	Protein translocase subunit SecD	2.0
oxaA PA5568	Putative protein OxaA	3.3
secG PA4747	Protein-export protein SecG	7.3
secA PA4403	Protein translocase subunit SecA	10.2
clpV1 PA0090	Protein ClpV1	2.5
ftsY PA0373	Signal recognition particle receptor FtsY	5.5
atpF PA5558	ATP synthase subunit b	8.5
atpA PA5556	ATP synthase subunit alpha	10.8
atpD PA5554	ATP synthase subunit beta	18.3
atpG PA5555	ATP synthase gamma chain	4.8
atpC PA5553	ATP synthase epsilon chain	2.0
zipA PA1528	Cell division protein ZipA	5.8
dacA	Penicillin-binding protein 5	5.8
fimV PA3115	Motility protein FimV	8.3
pctA PA4309	Chemotactic transducer PctA	4.2
PA2652	Putative chemotaxis transducer	2.2
PA1458	CheA homolog	3.3
PA4431	Putative Ubiquinol-cytochrome c reductase	3.3
ccoO1 PA1553	Cytochrome c oxidase	2.2
ccoP1 PA1552	Cytochrome c oxidase subunit	5.5
msbA PA4997	Lipid A export protein MsbA	2.8
gcd PA2290	Glucose dehydrogenase	7.5
ppiD PA1805	Peptidyl-prolyl cis-trans isomerase D	6.2
ftsH PA4751	Zinc metalloprotease FtsH	3.7
mexA PA0425	Multidrug resistance protein MexA	4.5
mexE PA2493	RND multidrug efflux protein MexE	15.8
pssA PA4693	Phosphatidylserine synthase	2.2
hflK PA4942	Protease subunit HflK	7.2
sdhA PA1583	Succinate dehydrogenase (A subunit)	14.2
sdhB PA1584	Succinate dehydrogenase (B subunit)	8.8
rho PA5239	Transcription termination factor Rho	2.0
typA PA5117	Regulatory protein TypA	2.5
lepA le PA0767	Elongation factor EF-4	3.0
PA4461	Putative ABC transporter	3.5
PA5258	Putative uncharacterized protein	2.2
PA3729	Putative uncharacterized protein	3.3
PA2873	Putative uncharacterized protein	3.3

Gene names	Protein names	SpC[b]
PA5528	Putative uncharacterized protein	2.7
accA PA3639	Acetyl-coenzyme A carboxylase	3.7
aceF aceB PA5016	Dihydrolipoyllysine-residue acetyltransferase	4.7
dnaJ PA4760	Chaperone protein DnaJ	8.3
gyrB PA0004	DNA gyrase subunit B	2.3
PA2840	Putative RNA helicase	2.0
mqo1 mqoA PA3452	Putative malate:quinone oxidoreductase 1	5.3
PA3262	Peptidyl-prolyl cis-trans isomerase	12.7
lon PA1803	Lon protease	2.0
clpX PA1802	Clp protease ClpX	3.7
ibpA PA3126	Heat-shock protein IbpA	2.3
pcnB PA4727	Poly(A) polymerase	4.3
PA0084	Putative uncharacterized protein	3.7
PA4438	Putative uncharacterized protein	2.5
PA3804	Putative uncharacterized protein	2.3
PA5146	Putative uncharacterized protein	2.3
PA4961	Putative uncharacterized protein	2.3
PA4842	Putative uncharacterized protein	2.3
PA4441	Putative uncharacterized protein	2.8
PA1592	Putative uncharacterized protein	2.7
PA0537	Putative uncharacterized protein	3.0
PA0126	Putative uncharacterized protein	2.0
pilA fimA PA4525	Pilin	6.0
ftsA PA4408	Cell division protein FtsA	3.2
tig PA1800	Trigger factor (TF)	17.8
mreB PA4481	Rod shape-determining protein MreB	5.5
PA4595	Putative ABC transporter	17.0
PA3019	Putative ABC transporter	3.7
PA1964	Putative ABC transporter	2.8
PA2735	Putative restriction-modification system protein	3.0
rpoA PA4238	RNA polymerase subunit alpha RpoA	4.7
rpoD rpoDA PA0576	RNA polymerase sigma factor RpoD	15.2
nusG PA4275	Transcription antitermination protein NusG	3.2
algP algR3 PA5253	Transcriptional regulatory protein AlgP	2.8
nusA PA4745	N utilization substance protein A NusA	2.0
rne PA2976	Ribonuclease E	45.5
rplJ PA4272	50S rP L10	6.5
rpmB PA5316	50S rP L28	5.0
rpsP PA3745	30S rP S16	2.7
rplE PA4251	50S rP L5	10.5
rpsK PA4240	30S rP S11	2.8
rpsD PA4239	30S rP S4	7.2
rpsL PA4268	30S rP S12	5.8
rpsC PA4257	30S rP S3	18.7
rpsB PA3656	30S rP S2	20.8

Gene names	Protein names	SpC[b]
tsf PA3655	Elongation factor EF-Ts	2.2
infB PA4744	Translation initiation factor IF-2	19.3
rpsA PA3162	30S rP S1	23.8
rpsH PA4249	30S rP S8	4.0
rpsE PA4246	30S rP S5	8.7
alaS PA0903	Alanyl-tRNA synthetase AlaS	2.2
aspS PA0963	Aspartyl-tRNA synthetase AspS	3.0

[a]For details see: Material and Methods - “Statistical analysis of MudPIT data”.

[b]Calculated as described in Material and Methods – “MudPIT analysis”.
