# Supplementary material for: Analysis of Pseudomonas aeruginosa Cell Envelope Proteome by Capture of Surface-Exposed Proteins on Activated Magnetic Nanoparticles
Source: PLoS One. 2012 Nov 30;7(11):e51062. doi: 10.1371/journal.pone.0051062 (PMC3511353; doi:10.1371/journal.pone.0051062)
Supplement: Table S2 — List of proteins (NP-CbP) identified by the denaturant treatment of NP-Env and considered bound by NPs at the cell surface because of the corresponding average Spectral Count (SpC) that resulted significantly higher by G-test (P>95%) than the SpC determined with the “shedding” control NP-Shed. SpC was calculated from the results of 4 MudPIT analyses. (PDF) [file pone.0051062.s005.pdf]

Table S2 List of proteins (NP-CbP) identified by the denaturant treatment of NP-Env and considered bound by NPs at the cell surface because of the corresponding average Spectral Count (SpC) that resulted significantly higher by G-test (P>95%) than the SpC determined with the “shedding” control NP-Shed[a]. SpC was calculated from the results of 4 MudPIT analyses.

Gene Name	Protein name	SpC[b]
PA3988	Putative uncharacterized protein	4.0
oprI PA2853	Major OM lipoprotein OprI	5.0
lptD imp ostA PA0595	LPS-assembly protein LptD	16.0
icmP PA4370	Insulin-cleaving metalloproteinase OM protein	2.0
pilQ PA5040	Fimbrial assembly protein PilQ	4.0
fpvA PA2398	Ferripyoverdine receptor	5.0
foxA PA2466	Ferrioxamine receptor FoxA	2.0
PA1271	Putative tonB-dependent receptor	2.0
oprH PA1178	OM protein H1 PhoP/Q	12.0
pal oprL PA0973	Peptidoglycan-associated lipoprotein OprL	29.0
oprF PA1777	OM porin F OprF	231.0
oprE PA0291	Anaerobically-induced OM porin OprE	2.0
PA1041	Putative OM protein	5.0
fliD PA1094	B-type flagellar hook-associated protein 2	2.0
pctA PA4309	Chemotactic transducer PctA	2.0
PA4431	Putative Ubiquinol-cytochrome c reductase	4.0
pagL PA4661	Lipid A 3-O-deacylase PagL	2.0
PA4423	Putative uncharacterized protein	2.0
PA3641	Putative amino acid permease	4.0
phaF PA5060	Polyhydroxyalkanoate synthesis protein PhaF	4.0
mexE PA2493	RND multidrug efflux protein MexE	8.0
PA0641	Putative bacteriophage protein	3.0
PA0833	Putative uncharacterized protein	34.0
PA2800	Putative uncharacterized protein	13.0
PA1053	Putative uncharacterized protein	6.0
fimV PA3115	Motility protein FimV	2.0
proB PA4565	Glutamate 5-kinase ProB	2.0
dnaJ PA4760	Chaperone protein DnaJ	2.0
PA3940	Putative DNA binding protein	10.0
rluB PA3179	Putative ribosomal pseudouridine synthase B	3.0
amrZ PA3385	Alginate and motility regulator Z AmrZ	2.0
PA3031	Putative uncharacterized protein	2.0
PA4639	Putative uncharacterized protein	4.0
PA0505	Putative uncharacterized protein	2.0
PA0070	Putative uncharacterized protein	8.0
ftsA PA4408	Cell division protein FtsA	2.0
pilA fimA PA4525	Pilin	2.0
mreB PA4481	Rod shape-determining protein MreB	3.0
PA4595	Putative ABC transporter	4.0
nusG PA4275	Transcription antitermination protein NusG	3.0

Gene Name	Protein name	SpC[b]
algP algR3 PA5253	Transcriptional regulatory protein AlgP	11.0
tsf PA3655	Elongation factor EF-Ts	2.0
infC PA2743	Translation initiation factor IF-3	11.0
rplW PA4261	50S rP L23	3.0
rplM PA4433	50S rP L13	9.0
rpsU PA0579	30S rP S21	7.0
rpsN PA4250	30S rP S14	4.0
rpsH PA4249	30S rP S8	4.0
rplR PA4247	50S rP L18	6.0
rplB PA4260	50S rP L2	44.0
rpmD PA4245	50S rP L30	8.0
rpmF PA2970	50S rP L32	3.0
rplJ PA4272	50S rP L10	3.0
rpmB PA5316	50S rP L28	13.0
rpsP PA3745	30S rP S16	4.0
rplS PA3742	50S rP L19	8.0
rpsQ PA4254	30S rP S17	3.0
rplE PA4251	50S rP L5	3.0
rpsK PA4240	30S rP S11	20.0
rpsD PA4239	30S rP S4	14.0
rpsL PA4268	30S rP S12	22.0
rpsC PA4257	30S rP S3	20.0
rpsB PA3656	30S rP S2	14.0

[a]For details see: Material and Methods - “Statistical analysis of MudPIT data”.

[b]Calculated as described in Material and Methods – “MudPIT analysis”.
