# Supplementary material for: Analysis of Pseudomonas aeruginosa Cell Envelope Proteome by Capture of Surface-Exposed Proteins on Activated Magnetic Nanoparticles
Source: PLoS One. 2012 Nov 30;7(11):e51062. doi: 10.1371/journal.pone.0051062 (PMC3511353; doi:10.1371/journal.pone.0051062)
Supplement: Table S3 — List of proteins identified by trypsin treatment of intact cells and considered “shaved” because of the corresponding average Spectral Count (SpC) that resulted significantly higher by G-test (P>95%) than the SpC determined in the absence of a trypsin treatment (“shedding” control). SpC was calculated from the results of 4 MudPIT analyses. (PDF) [file pone.0051062.s006.pdf]

Table S3 List of proteins identified by trypsin treatment of intact cells and considered “shaved” because of the corresponding average Spectral Count (SpC) that resulted significantly higher by G-test (P>95%) than the SpC determined in the absence of a trypsin treatment (“shedding” control)[a]. SpC was calculated from the results of 4 MudPIT analyses.

Gene name	Protein name	SpC[b]
oprF PA1777	OM porin F OprF	4.8
secG PA4747	Protein-export protein SecG	1.2
fba fda PA0555	Fructose-bisphosphate aldolase	1.0
sucB PA1586	Dihydrolipoyllysine-residue succinyltransferase SucB	3.7
fabG PA2967	3-oxoacyl-reductase FabG	1.2
lpxC envA PA4406	N-acetylglucosamine deacetylase LpxC	1.0
nrdB PA1155	ribonucleotide reductase NrdB	1.2
aceF aceB PA5016	Dihydrolipoyllysine-residue acetyltransferase	3.0
PA3031	Putative uncharacterized protein	1.2
PA0624	Putative uncharacterized protein	1.3
PA0623	Putative bacteriophage protein	1.7
PA0622	Putative bacteriophage protein	1.7
clpB PA4542	Chaperone protein ClpB	1.8
htpG PA1596	Chaperone protein htpG	7.0
tig PA1800	Trigger factor (TF)	8.8
PA3940	Putative DNA binding protein	1.7
hupB PA1804	HU beta subunit	2.5
nusA PA4745	N utilization substance protein A NusA	1.7
recA PA3617	RecA	2.5
rpoB PA4270	RNA polymerase subunit beta RpoB	1.7
rpoC PA4269	RNA polymerase subunit beta' RpoC	2.8
rpoD PA0576	RNA polymerase sigma factor RpoD	3.3
nusG PA4275	Transcription antitermination protein NusG	1.8
greA PA4755	Transcription elongation factor GreA	1.3
rne PA2976	Ribonuclease E	4.3
tsf PA3655	Elongation factor EF-Ts	1.0
infB PA4744	Translation initiation factor IF-2	2.3
efp PA2851	Elongation factor EF-P	1.0
rplB PA4260	50S rP L2	5.3
rplO PA4244	50S rP L15	3.3
rpmD PA4245	50S rP L30	1.2
rplJ PA4272	50S rP L10	2.0
rpmB PA5316	50S rP L28	1.5
rplM PA4433	50S rP L13	3.5
rpsI PA4432	30S rP S9	1.3
rpsU PA0579	30S rP S21	1.5
rpsA PA3162	30S rP S1	17.7
rpsJ PA4264	30S rP S10	3.3
rplI PA4932	50S rP L9	2.7
rplU PA4568	50S rP L21	1.0

Gene name	Protein name	SpC[b]
rplC PA4263	50S rP L3	3.3
rplP PA4256	50S rP L16	1.3
rpsQ PA4254	30S rP S17	1.2
rpsH PA4249	30S rP S8	2.7
rplR PA4247	50S rP L18	1.8
rpsM PA4241	30S rP S13	2.3
rplT PA2741	50S rP L20	1.0
rpsC PA4257	30S rP S3	2.2

[a]For details see: Material and Methods - “Statistical analysis of MudPIT data”.

[b]Calculated as described in Material and Methods – “MudPIT analysis”.
