# Supplementary material for: Analysis of Pseudomonas aeruginosa Cell Envelope Proteome by Capture of Surface-Exposed Proteins on Activated Magnetic Nanoparticles
Source: PLoS One. 2012 Nov 30;7(11):e51062. doi: 10.1371/journal.pone.0051062 (PMC3511353; doi:10.1371/journal.pone.0051062)
Supplement: Table S4 — Summary list of proteins that: i) were found following trypsin shaving of intact cells (Sh), ii) remained covalently bound to NPs after treatment of NP-Env with denaturants (NP-CbP) and iii) were identified after trypsin digestion of NP-Env (NP-EnP). (PDF) [file pone.0051062.s007.pdf]

Table S4. Summary list of proteins that: i) were found following trypsin shaving of intact cells (Sh), ii) remained covalently bound to NPs after treatment of NP-Env with denaturants (NP-CbP) and iii) were identified after trypsin digestion of NP-Env (NP-EnP).

**Gene name PA *locus***	**Protein name**	**Protein family**	**Function class[a]**	**Export signal[b]**	**Localization confidence (CC[c], class[d])**	**NP-CbP**	**NP-EnP**	**Sh**
oprF PA1777	OM porin F OprF	OmpA	1	I	OM,1 - P,1	+	+	+
pal oprL PA0973[e]	Peptidoglycan-associated lipoprotein OprL	OmpA	1	II	OM,1 - P,1	+	+	
icmP PA4370[e]	Insulin-cleaving metalloproteinase OM protein		1	II	OM,1	+	+	
oprI PA2853[e]	Major OM lipoprotein OprI		1	II	OM,1	+		
oprE PA0291	Anaerobically-induced OM porin OprE		1	I	OM,1 - P,1	+		
oprH PA1178	OM protein H1 PhoP/Q		1	I	OM,1 - P,1	+		
pilQ PA5040	Fimbrial assembly protein PilQ	GSP D	1	I	OM,1 - P,1	+		
pagL PA4661	Lipid A 3-O-deacylase PagL		1	I	OM,1 - P,1	+		
fpvA PA2398	Ferripyoverdine receptor	TonB-dependent receptor	1	I	OM,1 - P,1	+		
foxA PA2466	Ferrioxamine receptor FoxA	TonB-dependent receptor	1	I	OM, 1	+		
PA3988[e]	Putative uncharacterized protein		4	II	OM,1 - P,1	+		
lptD imp ostA PA0595	LPS-assembly protein LptD	LptD	2	I	OM,2 - P,1	+		
PA1041[e]	Putative OM protein	OmpA	3	II	OM,2	+		
PA0641	Putative bacteriophage protein		3		OM,2	+		
PA1271	Putative tonB-dependent receptor	TonB-dependent receptor	3	I	OM,2 - P,1	+		
PA2800	Putative uncharacterized protein		4	I	OM,2 - P,1	+		
PA0833[e]	Putative uncharacterized protein	OmpA	4	II	OM,2 - P,1	+		
PA1053[e]	Putative uncharacterized protein		4	II	OM,2	+		
PA3262[e]	Peptidyl-prolyl cis-trans isomerase	FKBP-type PPIase	3	II	OM,2		+	
PA0070[e]	Putative uncharacterized protein		2	II	P,1	+		
dacC PA3999	Penicillin-binding protein 5		2	I	P,2 - IM,2		+	
pilA fimA PA4525	Pilin	N-Me-Phe pilin	1	IV	E,3	+	+	
fliD PA1094	B-type flagellar hook-associated protein 2	FliD	1		F,1 - P,1	+		

Gene name PA *locus*	Protein name	Protein family	Function class[a]	Export signal[b]	Localization confidence (CC[c], class[d])	NP-CbP	NP-EnP	Sh
mexE PA2493[e]	RND multidrug efflux protein MexE		1	II	IM,3	+	+	
pctA PA4309	Chemotactic transducer PctA		1	I	IM,3	+	+	
PA4431	Putative Ubiquinol-cytochrome c reductase		3	I	IM,3	+	+	
PA3641	Putative amino acid permease		3		IM,3	+		
PA4423[e]	Putative uncharacterized protein		4	II	IM,3 - P,1	+		
mexA PA0425[e]	Multidrug resistance protein MexA	Membrane fusion protein	1	II	IM,1 - OM,1		+	
secD PA3821	Protein translocase subunit SecD	SecD/SecF	2	I	IM,2		+	
ftsH PA4751	Zinc metalloprotease FtsH	Peptidase M41	2	I	IM,2		+	
msbA PA4997	Lipid A export protein MsbA	ABC transporter	2		IM,2		+	
PA4461	Putative ABC transporter	ABC transporter	3		IM,2		+	
zipA PA1528	Cell division protein ZipA	ZipA	2		IM,3		+	
ppiD PA1805	Peptidyl-prolyl cis-trans isomerase D		2		IM,3 - P,1		+	
secG PA4747	Protein-export protein SecG	SecG	2		IM,3		+	+
rho PA5239	Transcription termination factor Rho		2		IM,3 - P,1		+	
atpF PA5558	ATP synthase subunit b	ATPase	2		IM,3		+	
sdhA PA1583	Succinate dehydrogenase (A subunit)		2		IM,3 - P,1		+	
sdhB PA1584	Succinate dehydrogenase (B subunit)		2		IM,3		+	
lepA le PA0767	Elongation factor EF-4		2		IM,3		+	
typA PA5117	Regulatory protein TypA		2		IM,3 - P,1		+	
pssA PA4693	Phosphatidylserine synthase		2		IM,3		+	
gcd PA2290	Glucose dehydrogenase		2	I	IM,3		+	
PA2652	Putative chemotaxis transducer		3	I	IM,3		+	
oxaA PA5568	Putative protein OxaA	OXA1/oxaA	4		IM,3		+	
PA5528	Putative uncharacterized protein		4	I	IM,3		+	
PA3729	Putative uncharacterized protein		4		IM,3		+	
PA2873	Putative uncharacterized protein		4		IM,3		+	
PA5258	Putative uncharacterized protein		4		IM,3		+	

Gene name<br>PA *locus*	Protein name	Protein family	Function class[a]	Export signal[b]	Localization confidence (CC[c], class[d])	NP-CbP	NP-EnP	Sh
fimV PA3115	Motility protein FimV		1	I	U,3	+	+	
PA4639[e]	Putative uncharacterized protein		4	II	U,3	+		
PA0505	Putative uncharacterized protein		4		U,3	+		
PA3031[e]	Putative uncharacterized protein		4	II	U,3	+		+
ccoP1 PA1552	Cytochrome c oxidase subunit		1		U,3		+	
hflK PA4942	Protease subunit HflK		2		U,3		+	
PA0537[e]	Putative uncharacterized protein		4	II	U,3		+	
PA4441	Putative uncharacterized protein		4		U,3		+	
PA1592[e]	Putative uncharacterized protein		4	II	U,3		+	
PA5146	Putative uncharacterized protein		4	I	U,3		+	
PA4961	Putative uncharacterized protein		4		U,3		+	
PA4842	Putative uncharacterized protein		4		U,3		+	
PA0126	Putative uncharacterized protein		4	II	U,3		+	
PA0624	Putative uncharacterized protein		4		U,3			+
PA0623	Putative bacteriophage protein		3		U,3			+
PA0622	Putative bacteriophage protein		3		U,3			+
rpsH PA4249	30S rP S8		2		C,1	+	+	+
algP algR3 PA5253	Transcriptional regulatory protein AlgP		1		C,3 - U,3	+	+	
tsf PA3655	Elongation factor EF-Ts		2		C,3 - P,1	+	+	+
nusG PA4275	Transcription antitermination protein NusG		2		C,3	+	+	+
rplJ PA4272	50S rP L10		2		C,3	+	+	+
rpmB PA5316	50S rP L28		2		C,3	+	+	+
rpsC PA4257	30S rP S3		2		C,3	+	+	+
rpsB PA3656	30S rP S2		2		C,3,P,1	+	+	
rpsP PA3745	30S rP S16		2		C,3	+	+	
rpsL PA4268	30S rP S12		2		C,3	+	+	
rpsK PA4240	30S rP S11		2		C,3	+	+	

Gene name PA *locus*	Protein name	Protein family	Function class[a]	Export signal[b]	Localization confidence (CC[c], class[d])	NP-CbP	NP-EnP	Sh
rpsD PA4239	30S rP S4		2		C,3	+	+	
rplE PA4251	50S rP L5		2		C,3	+	+	
ftsA PA4408	Cell division protein FtsA		2		C,3	+	+	
dnaJ PA4760	Chaperone protein DnaJ		2		C,3	+	+	
mreB PA4481	Rod shape-determining protein MreB		2		C,3	+	+	
PA4595	Putative ABC transporter	ABC transporter	3		C,3 - P,1	+	+	
amrZ PA3385	Alginate and motility regulator Z AmrZ		1		C,1	+		
infC PA2743	Translation initiation factor IF-3		2		C,1	+		
rplM PA4433	50S rP L13		2		C,1	+		+
rpsU PA0579	30S rP S21		2		C,1	+		+
rplR PA4247	50S rP L18		2		C,1	+		+
rplW PA4261	50S rP L23		2		C,1	+		
rpsN PA4250	30S rP S14		2		C,1	+		
phaF PA5060	Polyhydroxyalkanoate synthesis protein PhaF		2		C,1	+		
PA3940	Putative DNA binding protein		3		C,1	+		+
proB PA4565	Glutamate 5-kinase ProB		2		C,2	+		
rpsQ PA4254	30S rP S17		2		C,3	+		+
rplB PA4260	50S rP L2		2		C,3	+		+
rpmD PA4245	50S rP L30		2		C,3	+		+
rplS PA3742	50S rP L19		2		C,3	+		
rpmF PA2970	50S rP L32		2		C,3	+		
rluB PA3179	Putative ribosomal pseudouridine synthase B		4		C,3	+		
alaS PA0903	Alanyl-tRNA synthetase AlaS		2		C,1 - P,1		+	
rpsA PA3162	30S rP S1		2		C,1 - P,1		+	+
rpsE PA4246	30S rP S5		2		C,1		+	
aspS PA0963	Aspartyl-tRNA synthetase AspS		2		C,2 - P,1		+	
lon PA1803	Lon protease		2		C,2		+	

Gene name PA *locus*	Protein name	Protein family	Function class[a]	Export signal[b]	Localization confidence (CC[c], class[d])	NP-CbP	NP-EnP	Sh
aceF aceB PA5016	Dihydrolipoyllysine-residue acetyltransferase		1		C,3 - P,1		+	+
rpoD PA0576	RNA polymerase sigma factor RpoD		1		C,3		+	+
ccoO1 PA1553	Cytochrome c oxidase		1		C,3		+	
ftsY PA0373	Signal recognition particle receptor FtsY		2		C,3		+	
mqo1 mqoA PA3452	Putative malate:quinone oxidoreductase 1		2	II	C,3		+	
PA0084	Putative uncharacterized protein		2		C,3		+	
ibpA PA3126	Heat-shock protein IbpA		2		C,3		+	
tig PA1800	Trigger factor (TF)		2		C,3 - P,1		+	+
nusA PA4745	N utilization substance protein A NusA		2		C,3 - P,1		+	+
infB PA4744	Translation initiation factor IF-2		2		C,3 - P,1		+	+
rne PA2976	Ribonuclease E		2		C,3		+	+
accA PA3639	Acetyl-coenzyme A carboxylase		2		C,3		+	
atpD PA5554	ATP synthase subunit beta	ATPase	2		C,3 - P,1		+	
atpA PA5556	ATP synthase subunit alpha	ATPase	2		C,3 - P,1		+	
clpX PA1802	Clp protease ClpX		2		C,3		+	
gyrB PA0004	DNA gyrase subunit B		2		C,3 - P,1		+	
secA PA4403	Protein translocase subunit SecA	SecA	2		C,3		+	
clpV1 PA0090	Protein ClpV1		2		C,3		+	
pcnB PA4727	Poly(A) polymerase		2		C,3		+	
atpG PA5555	ATP synthase gamma chain	ATPase	2		C,3		+	
atpC PA5553	ATP synthase epsilon chain	ATPase	2		C,3		+	
rpoA PA4238	RNA polymerase subunit alpha RpoA		2		C,3		+	
PA3019	Putative ABC transporter	ABC transporter	3		C,3		+	
PA1964	Putative ABC transporter		3		C,3		+	
PA1458	CheA homolog		3		C,3		+	
PA2735	Putative restriction-modification system protein		3		C,3		+	
PA2840	Putative RNA helicase		3		C,3		+	

Gene name<br>PA *locus*	Protein name	Protein family	Function class[a]	Export signal[b]	Localization confidence (CC[c], class[d])	NP-CbP	NP-EnP	Sh
PA3804	Putative uncharacterized protein		4		C,3 - U,3		+	
PA4438	Putative uncharacterized protein		4		C,3		+	
recA PA3617	RecA		1		C,1 - P,1			+
hupB PA1804	HU beta subunit		1		C,1			+
rpsM PA4241	30S rP S13		2		C,1			+
fabG PA2967	3-oxoacyl-reductase FabG		2		C,2 - P,1			+
nrdB PA1155	ribonucleotide reductase NrdB		1		C,3 - P,1			+
lpxC envA PA4406	N-acetylglucosamine deacetylase LpxC		1		C,3 - P,1			+
clpB PA4542	Chaperone protein ClpB		2		C,3 - P,1			+
htpG PA1596	Chaperone protein htpG		2		C,3 - P,1			+
efp PA2851	Elongation factor EF-P		2		C,3 - P,1			+
sucB PA1586	Succinyltransferase SucB		2		C,3 - P,1			+
greA PA4755	Transcription elongation factor GreA		2		C,3 - P,1			+
rpoB PA4270	RNA polymerase subunit beta RpoB		2		C,3 - P,1			+
rpoC PA4269	RNA polymerase subunit beta' RpoC		2		C,3			+
fba fda PA0555	Fructose-bisphosphate aldolase		2		C,3			+
rpsI PA4432	30S rP S9		2		C,3			+
rplO PA4244	50S rP L15		2		C,3			+
rpsJ PA4264	30S rP S10		2		C,3			+
rplI PA4932	50S rP L9		2		C,3 - P,1			+
rplU PA4568	50S rP L21		2		C,3			+
rplC PA4263	50S rP L3		2		C,3			+
rplP PA4256	50S rP L16		2		C,3			+
rplT PA2741	50S rP L20		2		C,3			+

[a]For each protein, functional class is indicated according to annotations in Pseudomonas Genome Database (www.pseudomonas.com) [1]. **Class 1**: Function experimentally demonstrated in *P. aeruginosa*; **Class 2**: Function of highly similar gene experimentally demonstrated in another organism; **Class 3**: Function proposed based on presence of conserved amino acid motif, structural feature or limited sequence similarity to an experimentally studied gene. **Class 4**: Homologs of previously reported genes of unknown function, or no similarity to any previously reported sequences.

[b]Predicted export signal type according to the annotations in Pseudomonas Genome Database (www.pseudomonas.com) [1].

[c]**CC**: Cell compartment. **OM**: outer membrane; **P**: periplasm; **E**: extracellular; **F**: flagellar; **IM**: inner membrane; **U**: unknown; **C**: cytoplasmic.

[d]For each protein, localization confidence is indicated according to the annotations in Pseudomonas Genome Database (www.pseudomonas.com) [1]. **Class 1**: Subcellular localization experimentally demonstrated in *P. aeruginosa*; **Class 2**: Subcellular localization of highly similar gene experimentally demonstrated in another organism or to a paralog experimentally demonstrated in the same organism. BLAST expected value of 10^{-10} for query within 80-120% of subject length. **Class 3**: Subcellular localization computationally predicted by PSORT.

[e]Lipoprotein, known or predicted [2].

References

1. Winsor GL, Lam DK, Fleming L, Lo R, Whiteside MD, et al. (2011) Pseudomonas Genome Database: improved comparative analysis and population genomics capability for *Pseudomonas* genomes. Nucleic Acids Res 39: D596-600.
2. Remans K, Vercammen K, Bodilis J, Cornelis P (2010) Genome-wide analysis and literature-based survey of lipoproteins in *Pseudomonas aeruginosa*. Microbiology 156: 2597-2607.
